# Concurrent observation and physical practice facilitates early closed motor skill learning: evidence from EEG source functional connectivity and behavioral performance

**DOI:** 10.3389/fpsyg.2026.1865823

**Published:** 2026-09-18

**Authors:** Chen Guo, Yali Long, Chuandong Wei, Zhenyi Nie, Yueting Zhang, Yang Li, Changjiang Liu, Bing Shi

**Affiliations:** 1 Department of Physical Education, Xi'an Jiaotong University, Xi'an, China; 2 Sports Learning Science Lab, School of Physical Education, Shaanxi Normal University, Xi'an, China; 3 Weinan Vocational and Technical College, Weinan, China; 4 Qilu Institute of Technology, Jinan, China; 5 Neurology Department, Pucheng County Hospital, Weinan, China

**Keywords:** 24-style Tai Chi, closed motor skill, EEG, learning methods, observation, practice, source functional connectivity

## Abstract

**Background:**

Motor observation (MO) and concurrent observation and physical practice (MP) are two commonly used approaches during early motor skill acquisition. This study tests an integrated Action Observation Training-Neuroplasticity Theory (AOT-NPT) framework, hypothesizing that: (H1) MP will elicit stronger functional connectivity in frontoparietal networks compared to MO; (H2): transitioning from an easier to a more demanding motor task will be associated with changes in functional connectivity patterns, particularly in the MP condition; (H3): functional connectivity will demonstrate method-dependent relationships with behavioral performance, with MP expected to show stronger positive brain–behavior associations.

**Methods:**

A two-factor mixed design was employed. The between-subjects factor was learning methods (MO, MP), and the within-subjects factor was task difficulty (Easy, Medium). Mobile EEG recorded neural activity during 24-Style Tai Chi learning (MO vs. MP; easy vs. medium). Source-level connectivity analysis and video-based motor performance scoring were applied.

**Findings:**

Both EEG and behavioral data provided evidence that MP surpasses MO, as indicated by stronger source functional connectivity, better motor performance, and changes in functional connectivity during the transition from an easier to a more demanding task. Additionally, functional connectivity in MP was significantly positively correlated with motor performance.

**Contribution:**

The present findings support the neurobehavioral advantage of integrating physical practice into action observation during early closed motor skill learning. This validates the AOT-NPT framework and establishes a concurrent observation and physical practice model for closed motor skill pedagogy.

## 1 Introduction

The acquisition of complex motor skills relies on the integration of sensory information, cognitive processes, and motor execution within sensorimotor systems. Compared to motor execution, the cognitive processes underlying motor skill learning remain relatively understudied. While observation and physical practice are foundational learning methods in sports pedagogy, the neurocognitive mechanisms underlying these methods—particularly in closed motor skill acquisition—remain incompletely understood. This study bridges this gap by investigating how combining observation with physical practice modulates cortical functional connectivity during the learning of 24-Style Tai Chi, a standardized closed motor skill that requires precise multi-limb coordination. 24-Style Tai Chi provides a well-suited paradigm for neurocognitive research, and exploring its learning process will help elucidate the involvement of multiple brain regions during real-time learning, an area that remains underexplored in the literature. Beyond sensory and motor mechanisms, the role of bodily engagement in learning has received increasing attention from embodied cognition perspectives. Wilson (2002) highlighted the close relationship between cognition, bodily experiences, and action processes, while Skulmowski and Rey (2018) proposed an embodied learning taxonomy based on bodily engagement and task integration. From this perspective, motor observation and physical practice represent different learning conditions, with physical practice involving greater bodily engagement and action-related integration.

Studies on sports observation have primarily focused on observing pictures or video clips (Rominger et al., 2021), with little attention paid to real-time movements. While observation and motor imagery have been extensively studied, physical practice—a critical component of complex motor learning—remains underexplored from a neurocognitive perspective, as does its combination with observation. Previous evidence has highlighted both shared neural substrates and complementary behavioral effects. Regarding shared neural substrates, converging evidence indicates that observation and practice activate overlapping cortical regions (Avanzini et al., 2012). Numerous brain imaging studies have indicated that the frontal, parietal, and pre-motor areas active during action execution are also active during action observation (Caspers et al., 2010; Hardwick et al., 2018). This phenomenon is attributed to the mirror neuron system, which transforms observed sensory information into motor representations. TMS (Transcranial Magnetic Stimulation) studies have further demonstrated that observing specific motor acts selectively enhances motor evoked potentials in the muscles responsible for executing those same acts (Fadiga et al., 1995), suggesting a direct link between observed actions and motor preparation. For instance, observing chopstick manipulation increases motor-evoked potentials in hand muscles (Bazzini et al., 2023).

Despite these shared mechanisms, pure observation exhibits critical constraints. A study showed that observation alone was incapable of inducing alterations comparable to practice. For example, 7 min of movement observation alone proved insufficient to induce a measurable cortical reorganization (Zhang et al., 2011). Larssen et al. (2021) stated that observation does not appear to activate the same implicit learning processes as physical practice, instead, it seems to be more strategic in nature. Similarly, Trempe et al. (2011) argue that without movement production during observation, an asymptote cannot be reached. Consequently, observation alone may fail to provide sufficient stimulation to drive plastic reorganization in M1 (Primary motor cortex).

Emerging evidence underscores the superiority of combining observation and execution. From an embodied learning perspective, the advantage of combining observation and practice may arise from the increased bodily engagement and stronger task integration provided by physical execution. Unlike observation alone, physical practice allows learners to continuously integrate visual information with proprioceptive and kinesthetic feedback, thereby facilitating the refinement of motor representations. AOT (Action Observation Training) integrates observation and physical practice strategies, capitalizing on the mirror system's capacity to bind sensory and motor representations. In knot-tying tasks, AOT groups achieved 42% performance gains—exceeding the additive effects of isolated observation (10%) and practice (25%) (Bazzini et al., 2022). In a recent review by Rizzolatti et al. (2021) the neurophysiological evidence of AOT's role in promoting recovery, maintenance, and the acquisition of motor skills was reported. AOT enhances the proper motor program (i.e., the series of cortical and subcortical activations leading to the execution of a given action) across pre-motor and parietal areas. The review by Rudisch et al. (2024) also indicates that observation combined with practice has an advantage over isolated methods. The beneficial effect of the combined method may be due to the integration of both explicit and implicit learning strategies. Despite these advances, AOT applications to real-world closed motor skills remain constrained by three fundamental gaps: (1) Task Complexity Gap. Current AOT paradigms pre-dominantly employ simplified tasks (e.g., knot-tying) that lack the multi-limb coordination demands of real-world skills like Tai Chi; (2) Neurodynamic Measurement Gap. fMRI-based studies sacrifice movement authenticity for imaging feasibility, focus on learning results rather than process; meanwhile, behavioral metrics fail to capture real-time neuroplastic changes during learning; (3) Neurobehavioral Mechanism Gap. There remains limited understanding of how distinct learning methods shape the relationship between cortical network reorganization and behavioral outcomes.

Our study bridges these gaps by: (1) Introducing 24-Style Tai Chi as an ecologically valid model requiring continuous whole-body coordination and explicit difficulty stratification; (2) Employing mobile EEG (Electroencephalogram) source connectivity to decode cortical dynamics during naturalistic movement execution. EEG has the advantages of non-invasiveness, low cost, high temporal resolution, portability, and is increasingly employed for brain connectivity studies (Hassan et al., 2014). Recent studies have utilized functional connectivity as a metric for assessing sports learning. Rubino et al. (2024) found that stronger brain connectivity is correlated with more significant changes in motor sequence learning. On the basis of EEG, traceability technology has become a widely used tool that can identify functional networks associated with motor learning in the neocortex (Dattola et al., 2020). Unlike scalp-level coherence, this method isolates true directional interactions between cortical sources by accounting for volume conduction artifacts (Pascual-Marqui, 2007). An sLORETA (standardized low resolution brain electromagnetic tomography) analysis of table tennis athletes revealed that the advantage in temporal duration perception is closely related to the activation of the pre-frontal area and other areas, as well as the enhanced functional connectivity between the pre-frontal area and the parietal area, etc. (Song et al., 2024). Li et al. (2020) by employing EEG source space imaging analysis, proposed a wrapped time-frequency combined selection in the source domain to decode motor imagery tasks. Previous work has demonstrated that functional connectivity at the cortical source level is associated with visuo-motor skill learning (Manuel et al., 2018). Zhang et al. (2020) used lagged non-linear connectivity to calculate functional connectivity between pairs of ROIs (regions of interest), and demonstrated that this indicator captures changes in functional connectivity. Based on previous studies, we adopted lagged non-linear connectivity to evaluate functional connectivity among 20 ROIs (motor-learning-related brain regions referred to in the literature) (Berchicci et al., 2017) during the process of closed motor learning; (3) Establishing method-specific brain-behavior relationships by correlating functional connectivity patterns with quantitative motor performance. Therefore, this study aims to bridge critical gaps in current research—specifically in the neural representation of complex motor tasks, real-time neurodynamics during naturalistic learning, and method-dependent neurobehavioral coupling mechanisms. Integrating the AOT framework with Neuroplasticity Theory (NPT), we propose three exploratory hypotheses: H1: neural differentiation of learning methods. MP (concurrent observation and physical practice) will elicit stronger functional connectivity in frontoparietal networks compared to MO (motor observation); H2: task-level-related neural adaptation. Transitioning from an easier to a more demanding motor task will be associated with changes in functional connectivity patterns, particularly in the MP condition; H3: learning-method dependent neurobehavioral coupling. Functional connectivity will demonstrate method-dependent relationships with behavioral performance, with MP expected to show stronger positive brain–behavior associations. This study mainly investigates the following when learning a novel closed motor skill (24-Style Tai Chi): (1) the differences in source functional connectivity and motor performance between learning methods (observation and practice); (2) between task levels (easy and medium); and (3) the correlation between source functional connectivity and motor performance across different learning methods.

## 2 Theoretical integration framework

### 2.1 AOT mechanisms for sensorimotor integration

The Action Observation Training framework builds upon the mirror neuron system's proposed role in mapping observed actions onto motor representations. As established in Section 1, this system is associated with shared frontoparietal networks involved in both action execution and observation, supporting sensorimotor integration. Crucially, combining physical practice with observation (as in MP) may engage this mechanism more effectively than pure observation (MO), as evidenced by supra-additive behavioral gains in knot-tying tasks (42 vs. 10%). This provides a theoretical basis for predicting neurocognitive differences between learning methods (H1).

### 2.2 Neuroplasticity theory as the substrate for learning-induced changes

NPT posits that motor learning induces cortical reorganization through experience-dependent mechanisms. Two core principles are central to our hypotheses: (1) Use-dependent plasticity: sustained motor practice can induce reorganization of relevant motor cortical regions, thereby contributing to improved motor control. Studies have shown enhanced sensorimotor experiences and motor skill observation can facilitate motor learning. This suggests that combining observation with practice may facilitate learning-related neural adaptations (Mcgregor et al., 2018).

This principle predicts that MP's repeated sensorimotor experiences may contribute to stronger connectivity changes than MO (H1); (2) Adaptive plasticity: increasing task demands may be associated with connectivity reorganization. This principle predicts that elevating task complexity will induce measurable reorganization in functional connectivity patterns (H2), particularly in the MP condition.

### 2.3 Integrated framework for brain-behavior relationships

The AOT-NPT integration provides a theoretical basis for investigating connectivity–behavior relationships. Empirical AOT studies have generally demonstrated that combining practice with observation yields superior behavioral outcomes compared to observation-only approaches. Complementarily, NPT provides a framework for understanding how such integrated training may induce learning-related neural adaptations in the motor system. Crucially, this evidence chain leads to a specific, testable prediction: enhanced functional connectivity in MP should positively correlate with behavioral learning outcomes (H3).

## 3 Materials and methods

A 2 × 2 factorial mixed-design experiment was conducted. The between-subjects factor comprised two methods: MO and MP. The within-subjects factor involved two levels of task difficulty: easy task (ET) and medium task (MT).

### 3.1 Participants

Participants were recruited with the following inclusion criteria. (1) College students with no prior experience in 24-Style Tai Chi. The rationale for recruiting only beginners was to control for varying student experiences, thereby minimizing their impact on motor performance and learning-related differences. (2) Right-handed students, as assessed by the Edinburgh Handedness Inventory (Oldfield, 1971). As depicted in our analysis flowchart (Figure 1a), a total of 45 college students were initially recruited. Data from two students were excluded due to poor data quality and recording issues. Consequently, 43 volunteers were included in this study. Participants were randomly assigned to two groups. The MP group comprised 21 participants (seven males, mean age = 20.90 years, SD = 2.28 years). The MO group included 22 participants (eight males, mean age = 24.55 years, SD = 3.95 years). A chi-square test confirmed no gender bias among the groups (χ^2^ = 0.044, *P* = 0.833). The sample size was calculated *a priori* using G^*^Power 3.1 (Faul et al., 2007) software (Cohen's *d* = 0.25, α = 0.05, power = 0.8) (Zuo et al., 2021), indicating that the sample size was adequate. All participants volunteered and were compensated with 30 RMB and souvenirs. Informed consent was obtained prior to the experiment. This study protocol was approved by Shaanxi Normal University Academic Committee (NO. 202412050) and complied with ethical standards outlined by the 1964 Helsinki Declaration and its subsequent amendments or comparable ethical standards.

**Figure 1:**
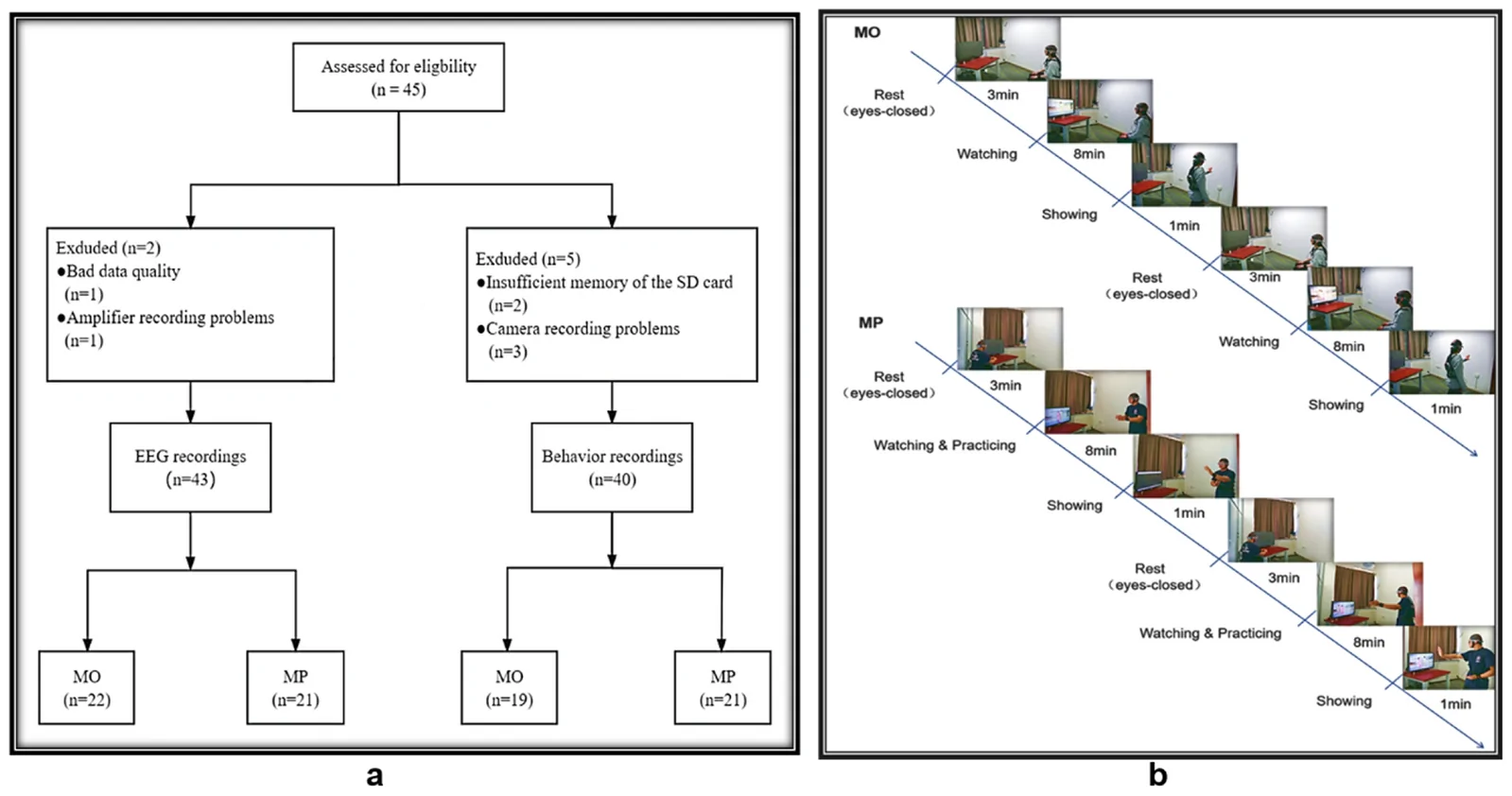
**(a)** Flow chart of the analysis sample. **(b)** Experimental procedure of this study. For MO group, in the section of learning ET, participants initially rested in a sitting position for 3 min, subsequently watched the ET video for 8 min, and ultimately showed the ET motion for 1 min. In the section of learning MT, participants rested in the sitting position for 3 min, then watched the MT video for 8 min, and subsequently showed the MT motion for 1 min. For the MP group, in the stage of learning ET, participants initially rested in a sitting position for 3 min, then practiced ET while watching the ET video, and finally showed the ET motion for 1 min. In the stage of learning MT, participants rested in a sitting position for 3 min, then practiced MT while watching the MT video, and ultimately showed the MT motion for 1 min. MO: motor observation; MP: concurrent observation and physical practice.

### 3.2 Experiment materials and procedure

To investigate the learning process of realistic closed motor skills, we targeted sports characterized by stable postural control, relatively slow body-position transitions, limited vigorous movements, and less sweating. Finally, we selected 24-Style Tai Chi—a traditional Chinese exercise program—as our experiment tasks to study the neurocognitive mechanism of natural closed motor skill learning process. It is commonly taught across educational levels in China and has been widely employed in EEG and functional near-infrared spectroscopy (fNIRS) studies. Our experiment materials consisted of two 24-Style Tai Chi teaching videos sourced from the Chinese University MOOC platform (https://www.icourse163.org/). These two motions were selected from the 24-Style Tai Chi repertoire through expert evaluation based on specific criteria. Fifteen martial arts experts rated the 24 Tai Chi motions. These experts were qualified to evaluate and rank the 24-Style Tai Chi, providing a comprehensive basis for material selection (For demographic information about the martial arts experts and the selection process of experiment materials, please see Supplementary material S1). Ultimately, “Hold the Lute” was selected as the Easy Task (ET), and “High Pat on Horse” was chosen as the Medium Task (MT), as these two movements exhibited the lowest likelihood of inducing movement-related artifacts.

Participants were positioned 1.1m from an LCD monitor (PHILIPS, BDM4350, Netherlands) within a light- and sound-attenuated room. Initially, participants rested for 3 min, then learned the ET first, followed by a 1-min motor performance phase (demonstration phase for behavioral assessment, see Section 3.5). The second stage followed an identical procedure, except that participants learned MT. For the specific experimental procedure, please see Figure 1b. For participants in the MO group, they rested (eyes closed) and observed the instructional videos while seated, while MP participants rested seated and then stood for the practice stage. These arrangements aimed to minimize motor artifacts due to body position changes between different experiment steps. The LCD displayed the two Tai Chi instructional videos separately (ET: “Hold the Lute”; MT: “High Pat on Horse”). Participants in MP practiced the movements while simultaneously watching the instructional videos (“Watching & Practicing”), whereas participants in MO only observed without physical practice (Watching). To control for irrelevant variables, teaching videos were consistent across groups, with equalized learning durations. EEG data recording per participant took approximately 24 min (For the flowchart of this study, please see Figure 2).

**Figure 2:**
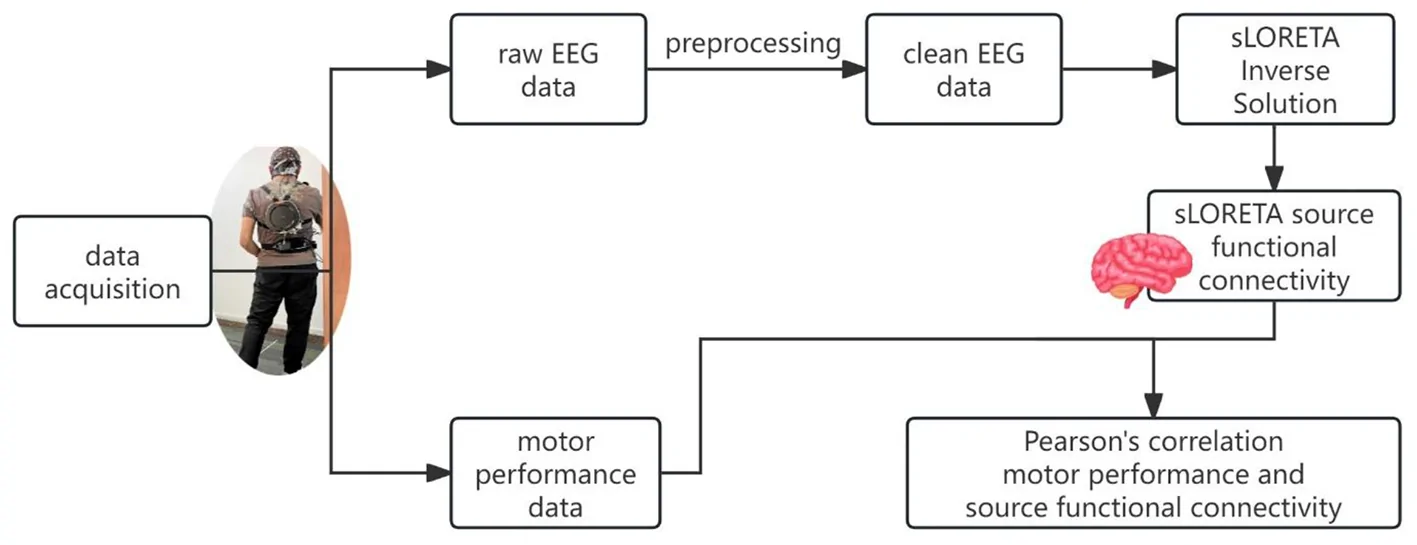
Flow chart of the study.

### 3.3 EEG data recording and pre-processing

EEG data were wirelessly recorded by a wearable EEG recording system (TMSi, SAGA 64+ DR, Netherlands) with a sampling rate of 1,000 Hz. Sixty-four electrodes were positioned according to the International 10–20 System, with impedance maintained below 10 kΩ. The data recorder was positioned on the participants' backs, and the docking station received and saved the EEG signals wirelessly to the experiment computer. The wireless system was adopted to minimize restrictions on body movement and enable EEG acquisition during Tai Chi performance.

MATLAB 2013b software (MathWorks, Natick, MA, USA) and EEGLAB (v13.0.0) toolbox were employed for data analysis. Bad electrodes (i.e., electrodes with large drift exceeding 1 min) were identified through visual inspection and interpolated using a spherical spline method (Perrin et al., 1989). EEG data were segmented into task-specific epochs based on manually annotated event markers recorded during the experiment. Drift-contaminated segments were removed and the remaining data were concatenated. Data were then average-referenced, resampled to 500Hz, and segmented into 2-s intervals. A high pass filter at 1 Hz, a low pass filter at 100 Hz, and a notch filter at 48–53Hz were applied.

Considering that participants in the MP condition performed whole-body Tai Chi movements during EEG acquisition, potential movement-related artifacts were carefully considered during pre-processing. Components associated with ocular, cardiac, muscular, swallowing, and other non-neural artifacts were manually identified and removed using Independent Component Analysis (ICA) (Jung et al., 2000). Extreme values exceeding 100 μV were rejected. The same pre-processing procedures and artifact rejection criteria were applied to both MO and MP conditions. (For details, see Supplementary material S4).

### 3.4 sLORETA-based non-linear lagged connectivity

Previous studies have pre-dominantly analyzed functional connectivity from head-surface signals; however, it is acknowledged that this electrode-space approach may not accurately infer the activity of the underlying brain regions due to volume conduction distortions (Painold et al., 2020). Volume conduction and reference-dependency make the interpretability of sensor-based connectivity measures difficult (Engel et al., 2013). Functional connectivity measures based on estimated intracortical signals, such as coherence, have been found to be biased and affected by low spatial resolution and volume conduction (Nolte et al., 2004). Therefore, EEG source imaging is a pre-requisite for functional connectivity analysis (He et al., 2019). To overcome these limitations and investigate key brain-regions connections in closed-skill motor learning, the present study performed functional connectivity analysis in source space. This method also allows this study to align signals with Brodmann areas, thus enabling ROI-based connectivity analysis that directly links to cortical functions (Michel C and Brunet, 2019). This study used the ROInonLinLagConn (non-linear lagged connectivity) index between 20 ROIs (Table 1), yielding 190 intracortical lagged connectivity values (20^*^19/2) for each participant and each frequency band. ROInonLinLagConn is related to non-linear similarity (a properly corrected phase-synchronization value), encompassing solely causal-physiological connectivity information. It is an inverse solution algorithm that localizes intracortical source signals from the head-surface recordings before calculating functional connectivity, thus remaining unaffected by the zero-lag artifact due to volume conduction (Perrin et al., 1989). Lagged connectivity has been applied in studies on individuals with schizophrenia and meditation, demonstrating reliable performance for connectivity estimation (Pascual-Marqui et al., 2011).

**Table 1:** The 20 ROIs defined in the current study.

ROI	*X*	*Y*	*Z*	BA	Brain regions	Abbreviations
1	−43	48	27	46	Dorsolateral pre-frontal cortex	DLPFC
2	0	59	37	10	Fronto-parietal cortex	FPC
3	34	57	27	10	Fronto-parietal cortex	FPC
4	−47	53	3	10	Fronto-parietal cortex	FPC
5	−24	67	21	10	Fronto-parietal cortex	FPC
6	20	71	16	10	Fronto-parietal cortex	FPC
7	49	52	8	46	Dorsolateral prefrontal cortex	DLPFC
8	−32	66	−6	10	Fronto-parietal cortex	FPC
9	4	71	−1	10	Fronto-parietal cortex	FPC
10	38	65	−3	10	Fronto-parietal cortex	FPC
11	−12	−9	78	6	Premotor cortex and supplementary motor area	PMC & SMA
12	−25	−27	75	4	Primary motor cortex	M1
13	6	−32	79	6	Premotor cortex and supplementary motor area	PMC & SMA
14	−12	−47	79	5	Somatosensory association cortex	SAC
15	−24	−58	73	7	Somatosensory association cortex	SAC
16	11	−52	77	5	Somatosensory association cortex	SAC
17	−12	−62	72	7	Somatosensory association cortex	SAC
18	−20	−77	60	7	Somatosensory association cortex	SAC
19	15	−75	63	7	Somatosensory association cortex	SAC
20	−11	−87	46	7	Somatosensory association cortex	SAC

BA, Brodmann area.

Studies have demonstrated that pre-frontal and front-parietal areas play central roles in motor sequence learning, visuo-motor behaviors, and task complexity processing (Berchicci et al., 2017). Therefore, the present study adopted a literature-guided ROI selection strategy, focusing on cortical regions that have been consistently implicated in motor learning. Although motor learning also involves other brain regions, the current study focused specifically on frontal and parietal cortical networks because these regions are consistently reported cortical substrates underlying motor skill acquisition and are suitable targets for EEG source-space connectivity analysis. Accordingly, 20 ROIs covering frontal, parietal, and sensorimotor regions were selected to investigate areas related to 24-Style Tai Chi learning. The selection of these ROIs was based on their established roles in motor control and learning. DLPFC (Dorsolateral pre-frontal cortex) is related to memory performance (Grady et al., 2010). FPC (frontoparietal cortex) and SAC (Somatosensory association cortex) are strongly associated with cognitive and motor functions (Koini et al., 2018). M1 and SMA (Supplementary motor area) have been shown to participate in preparation and execution during sequential movements (Swinnen, 2002). PMC (Premotor cortex) has been demonstrated to play a key role in sensorimotor integration (Martinez et al., 2014; Hardwick et al., 2013). The cortical ROIs were defined as detailed in Table 1, with their positions illustrated in Figure 3a.

**Figure 3:**
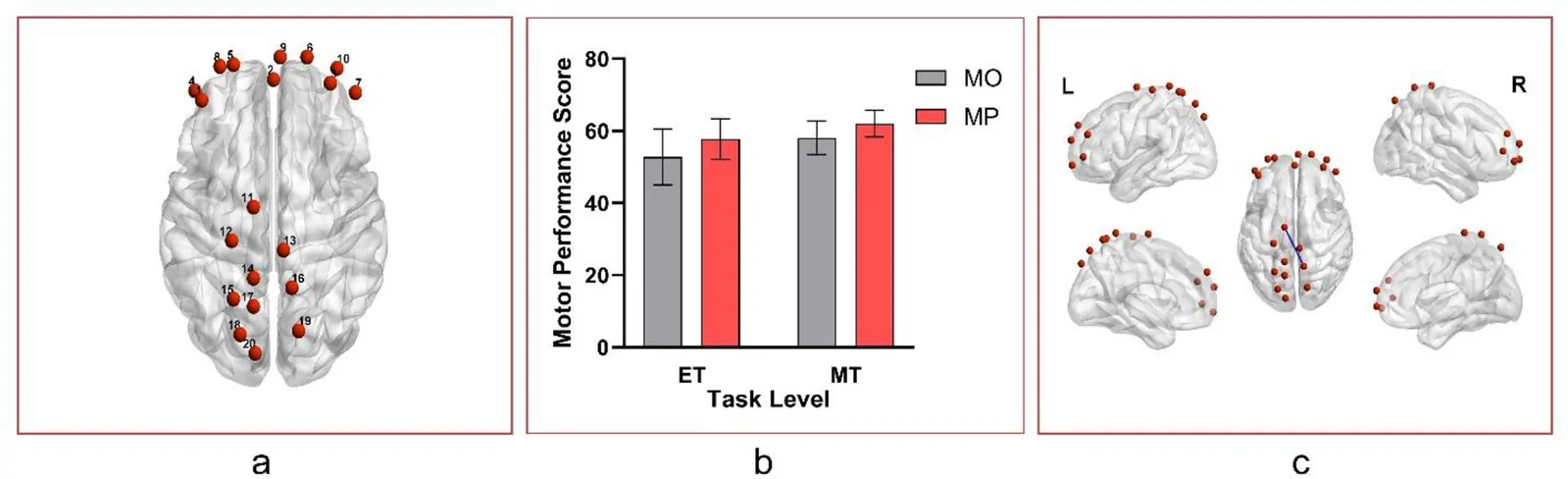
**(a)** Positions of the 20 ROIs of the study. **(b)** Motor performances of ET and MT in MO group and MP group. **(c)** Source functional connectivity differences between ET and MT in MP group on δ band. ET, easy task; MT, medium task; MO, motor observation; MP, concurrent observation and physical practice.

Although the selected ROIs can be anatomically grouped into several broader cortical regions, individual ROIs were not assumed to be functionally identical. Each ROI represented a distinct cortical source location defined by different Brodmann areas and hemispheric positions. Within the same anatomical region, different cortical subregions may exhibit distinct patterns of structural connectivity and functional involvement during motor learning. Therefore, connectivity analyses were performed at the level of individual ROIs rather than averaged anatomical regions, allowing the identification of specific network interactions associated with different learning methods.

The EEG data for connectivity analysis were extracted from the total data (“Watching” segment for MO, and “Watching & Practicing” for MP), as these phases were considered to reflect neural processes associated with motor skill acquisition. Scalp EEG signals from 62 electrodes were input to the sLORETA to estimate cortical source activity. Time-series of current density were extracted for the ROIs and 6 frequency bands: δ (1–4 Hz), θ (4–8 Hz), α (8–13 Hz), β (13–30 Hz), low-γ (30–48Hz), and high-γ (52–80Hz). Connectivity was computed between source-level ROI pairs using the ROInonLinLagConn metric. The bands were selected for their relevance in closed motor skill learning. Delta band activity has been associated with information related to movement initiation and direction. Theta band is related to cognitive load. Gamma band is associated with brain-muscle synchronization (Weibo, 2017), spatial memory consolidation (Kanta et al., 2024), and higher-order cognitive functions (Wiesman A and Wilson T, 2019). For example, Cheyne et al. (2008) identified gamma oscillations in the primary motor cortex during self-paced movements of the upper and lower limbs. Hassan et al. (2014) selected gamma band to investigate cognitive tasks performed by subjects.

### 3.5 Behavioral tests

Behavioral data for participants in MO were collected after each “Watching” section. Similarly, data for participants in MP were gathered following each “Watching & Practicing” section (refer to the “showing” section in Figure 1b). These data were used to evaluate the learning outcomes. Behavioral performance data were analyzed independently of the neural data. During the behavioral tests, students were asked to demonstrate the motion they had just learned in front of a digital video camera (Panasonic, AG-UX90MC, Japan) within 1 min following the learning phase. Out of 45 participants, behavioral data were obtained after all experiments concluded. Due to camera recording issues, data from 5 individuals were either damaged or incomplete, leading to their exclusion. Ultimately, 40 students' valid behavioral data were collected (as shown in Figure 1a). The recorded videos were anonymously packed for evaluation by three Tai Chi experts. The judges were blinded to group allocation and task condition. These judges, who were graduate students majoring in Traditional Ethnic Sports who also served as physical education instructors for 24-Style Tai Chi courses, were well-qualified to assess the participants' motor performance. Motor performance was evaluated using a standardized 24-Form Tai Chi performance scoring rubric (100-point scale), which assessed movement accuracy, technical execution, movement coordination, fluency, and completion of the routine. Detailed scoring criteria are provided in Supplementary material S1. Three judges independently evaluated each participant's motion by watching the demonstration video and assigned individual motor performance scores. For each motion, each participant received three motor performance scores. Subsequently, a final motor performance score for each motion and for each participant was calculated as the average of these three scores. Inter-rater reliability among the three judges was assessed using a two-way random-effects intraclass correlation coefficient with absolute agreement [ICC(2, k)]. Each participant had two motor performance scores: one for “Hold the Lute” (ET) and another for “High Pat on Horse” (MT).

### 3.6 Statistical analysis

All statistical procedures were implemented according to data characteristics and research design objectives. Given the exploratory nature of this study, all pre-defined ROIs and frequency bands were included in the connectivity analyses. For each frequency band, ROI-to-ROI source functional connectivity values from 190 connections were entered into permutation-based statistical analyses implemented within the sLORETA framework. Group comparisons (MO vs. MP) for each task and task comparisons (ET vs. MT) for each group were conducted. All reported between-group differences in functional connectivity were calculated as MP connectivity values minus MO values. Multiple comparisons among the 190 connections were controlled separately for each frequency band using the statistical non-parametric mapping (SnPM) approach implemented in sLORETA with 5,000 permutations. Statistical significance was determined based on corrected *p*-values with a threshold of *p* < 0.05. Therefore, the *p*-values reported in the Results section represent SnPM-corrected values rather than uncorrected *p*-values (for details, see Supplementary material S4.).

Behavioral data were analyzed using a two-way mixed-design analysis of variance (ANOVA) in SPSS 26.0 (IBM Corp., USA), with Learning Methods (MO vs. MP) as the between-subject factor and Task Levels (ET vs. MT) as the within-subject factor. Main effects of Learning Methods and Task Levels, as well as the Learning Methods × Task Levels interaction, were examined. All statistical tests were two-tailed with the significance level set at *p* < 0.05. To examine the potential influence of age differences between groups, separate ANCOVAs were also conducted for ET and MT motor performance, with age entered as a covariate.

For correlation analyses between source functional connectivity and behavioral performance, only participants with both valid EEG and behavioral datasets were included. Each participant's connectivity values were matched with their own motor performance scores for individual-level correlation analysis. Pearson correlation analyses were performed separately for the MO and MP groups to examine the relationships between motor performance scores and the source functional connectivity values within each frequency band. Correlation coefficients were calculated in MATLAB. All statistical tests were two-tailed. To account for multiple comparisons, the resulting *p*-values were corrected using the false discovery rate (FDR) procedure separately for each frequency band and each group. Statistical significance was set at *p* < 0.05 after FDR correction.

## 4 Results

### 4.1 Behavioral results

Inter-rater reliability among the three Tai Chi experts was moderate for ET (ICC = 0.705, 95% CI [0.419, 0.849], *F*_(38, 76)_ = 4.602, *p* < 0.001) and good for MT (ICC = 0.766, 95% CI [0.539, 0.879], *F*_(39, 78)_ = 5.651, *p* < 0.001). An independent-samples *t*-test was conducted to examine age differences between groups. Participants in the MO group were significantly older than those in the MP group [*t*(34) = 3.73, *p* < 0.001]. A 2 (Learning Method: MP vs. MO) × 2 (Task Level: ET vs. MT) mixed-design ANOVA was conducted, and a significant main effect of Learning Method was observed [F_(1, 37)_ = 7.491, *p* = 0.009, η*p*^2^ = 0.168], indicating that participants in the MP group achieved higher motor performance scores than those in the MO group (Figure 3b). No significant Learning Method × Task Level interaction was found [*F*(1, 37) = 0.490, *p* = 0.488, η*p*^2^ = 0.013], suggesting that the behavioral advantage of concurrent observation and physical practice over observation alone was comparable across the two motor tasks. A significant main effect of Task Level was also observed [*F*_(1, 37)_ = 44.248, *p* < 0.001, η*p*^2^ = 0.545]. Because task difficulty was confounded with task order in the present design, the task-level effect was interpreted cautiously and was not used as evidence for difficulty-related behavioral differences. Given the significant age difference between groups, separate ANCOVAs were further conducted for ET and MT performance with age entered as a covariate to examine whether the group effect remained after controlling for age. For ET performance, the main effect of learning method remained significant after controlling for age, *F*_(1, 37)_ = 6.770, *p* = 0.013, η*p*^2^ = 0.158, whereas the effect of age was not significant, *F*_(1, 37)_ = 1.381, *p* = 0.248. Similarly, for MT performance, the main effect of learning method remained significant after controlling for age, *F*_(1, 37)_ = 11.623, *p* = 0.002, η*p*^2^ = 0.239, while the covariate effect of age was not significant, *F*_(1, 37)_ = 1.647, *p* = 0.207. These results indicated that the advantage of MP over MO remained significant after controlling for age.

### 4.2 Comparison of source functional connectivity between different task levels

As shown in Figures 3b, 3c, comparison of source functional connectivity between ET and MT revealed that, in the MP group, source functional connectivity between ROI 11 (BA 6, PMC & SMA) and ROI 16 (BA 5, SAC) was significantly higher in MT than in ET in the δ band (*t* = 4.05, *P* = 0.047). No task difference was found in the MO group. Line plots showing the 190 connectivity values across frequency bands, groups (MO and MP), and tasks (ET and MT) are provided in Supplementary material S3.

### 4.3 Comparison of source functional connectivity in different learning methods

During ET learning, source functional connectivity in the MP group was significantly higher than in the MO group in the high-γ band between ROI 4 (BA 10, FPC) and ROI 12 (BA 4, M1; *t* = 3.76, *P* = 0.047), as well as between ROI 7 (BA 46, DLPFC) and ROI 19 (BA 7, SAC; *t* = 3.76, *P* = 0.047; Figure 4a).

**Figure 4:**
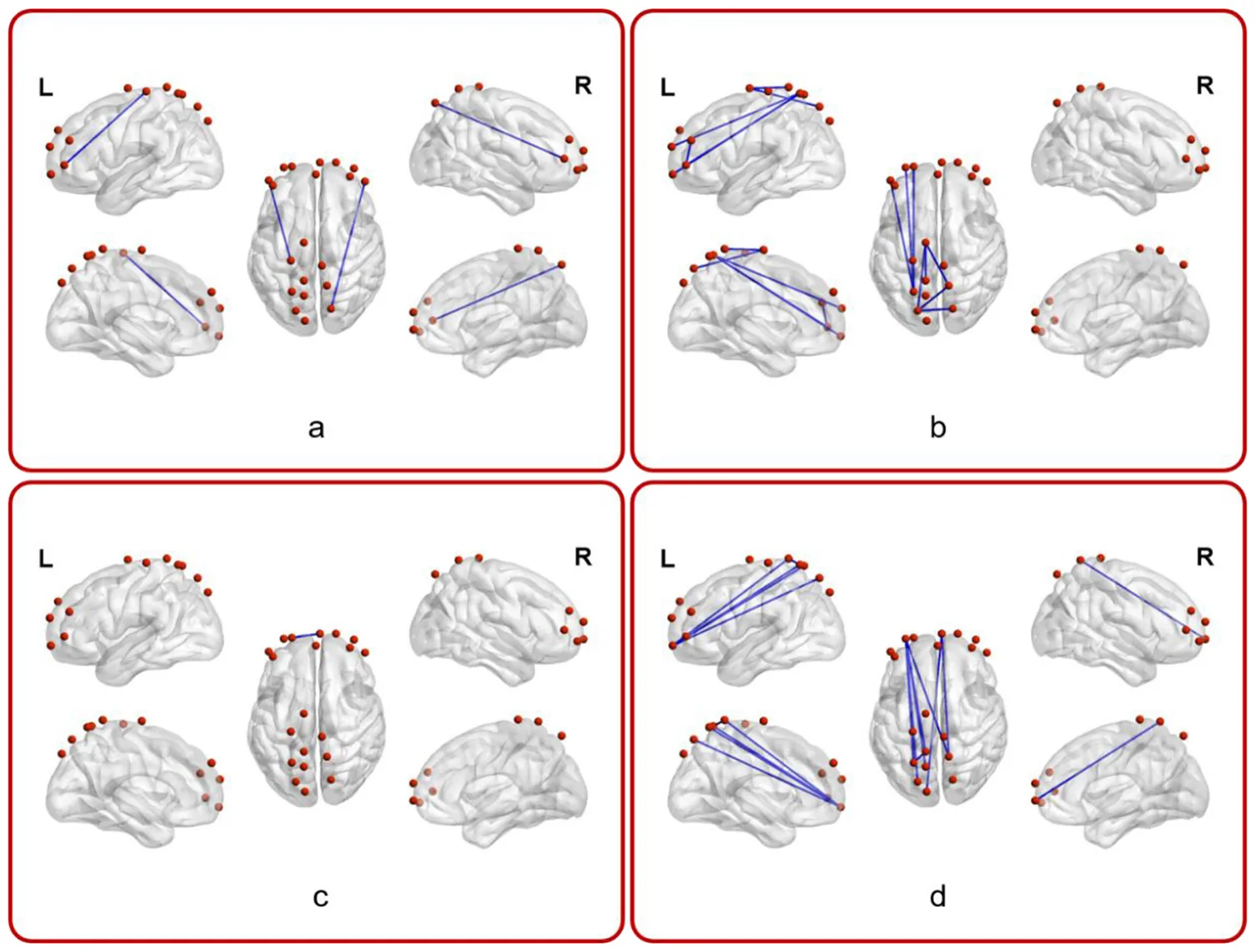
**(a)** During learning ET, the source functional connectivity differences between MO and MP on high-γ band. **(b)** During learning MT, the source functional connectivity differences between MO and MP on δ band. **(c)** During learning MT, the source functional connectivity differences between MO and MP on θ band. **(d)** During learning MT, the source functional connectivity differences between MO and MP on low-γ band. ET, easy task; MT, medium task; MO, motor observation; MP, concurrent observation and physical practice.

During MT learning, significant differences in functional connectivity were observed between MP and MO across the δ, θ, and low-γ frequency bands. In the δ band (Figure 4b), 9 ROI pairs' source functional connectivity in MP was significantly higher than that in MO, particularly involving interactions between ROI 1–ROI 4 (*t* = 3.24, *P* = 0.040), ROI 11–ROI 14 (*t* = 3.81, *P* = 0.037), ROI 4–ROI 15 (*t* = 3.53, *P* = 0.034), ROI 5–ROI 15 (*t* = 2.76, *P* = 0.042), ROI 8–ROI 15 (*t* = 3.07, *P* = 0.042), ROI 11–ROI 16 (*t* = 3.69, *P* = 0.034), ROI 11–ROI 18 (*t* = 3.78, *P* = 0.043), ROI 16–ROI 18 (*t* = 2.97, *P* = 0.040), ROI 18–ROI 19 (*t* = 3.39, *P* = 0.042). In the θ band (Figure 4c), MP exhibited significantly higher functional connectivity than MO specifically between two ROIs within the FPC: ROI 5 (BA 10) and ROI 9 (BA 10; *t* = 2.87, *P* = 0.041). In the low-γ band (Figure 4d), eight ROI pairs showed stronger connectivity in MP than in MO, primarily involving interactions between the ROI 8–ROI 14 (*t* = 2.74, *P* = 0.044), ROI 8–ROI 15 (*t* = 3.71, *P* = 0.036), ROI 14–ROI 15 (*t* = 3.77, *P* = 0.031), ROI 8–ROI 16 (*t* = 2.54, *P* = 0.036), ROI 9–ROI 16 (*t* = 3.20, *P* = 0.036), ROI 8–ROI 17 (*t* = 3.62, *P* = 0.043), ROI 8–ROI 18 (*t* = 3.45, *P* = 0.037), ROI 9–ROI 20 (*t* = 3.13, *P* = 0.046).

### 4.4 Correlations between source functional connectivity and motor performance in the MO and MP groups

As shown in Figure 5, ET-related source functional connectivity in MO was negatively correlated with ET motor performance in the δ, α, low-γ and high-γ bands (r range: −0.73 < r < −0.47, all *p* < 0.05). MT-related source functional connectivity in MO was negatively correlated with MT motor performance in the δ, θ, α, β and low-γ bands (r range: −0.65 < r < −0.48, all *p* < 0.05). In contrast, ET-related functional connectivity in MP was positively correlated with ET motor performance in the δ, α, β, low-γ, and high-γ bands (r range: 0.44 < r < 0.57, all *p* < 0.05; Figure 6). MT-related functional connectivity in the MP group was positively correlated with MT motor performance in the δ, α, β, and low-γ bands (r range: 0.45 < r < 0.66, all *p* < 0.05). Diagonal elements (self-connections) were excluded due to their invariable value (1) and lack of variability. (Full statistics: Supplementary material S2).

**Figure 5:**
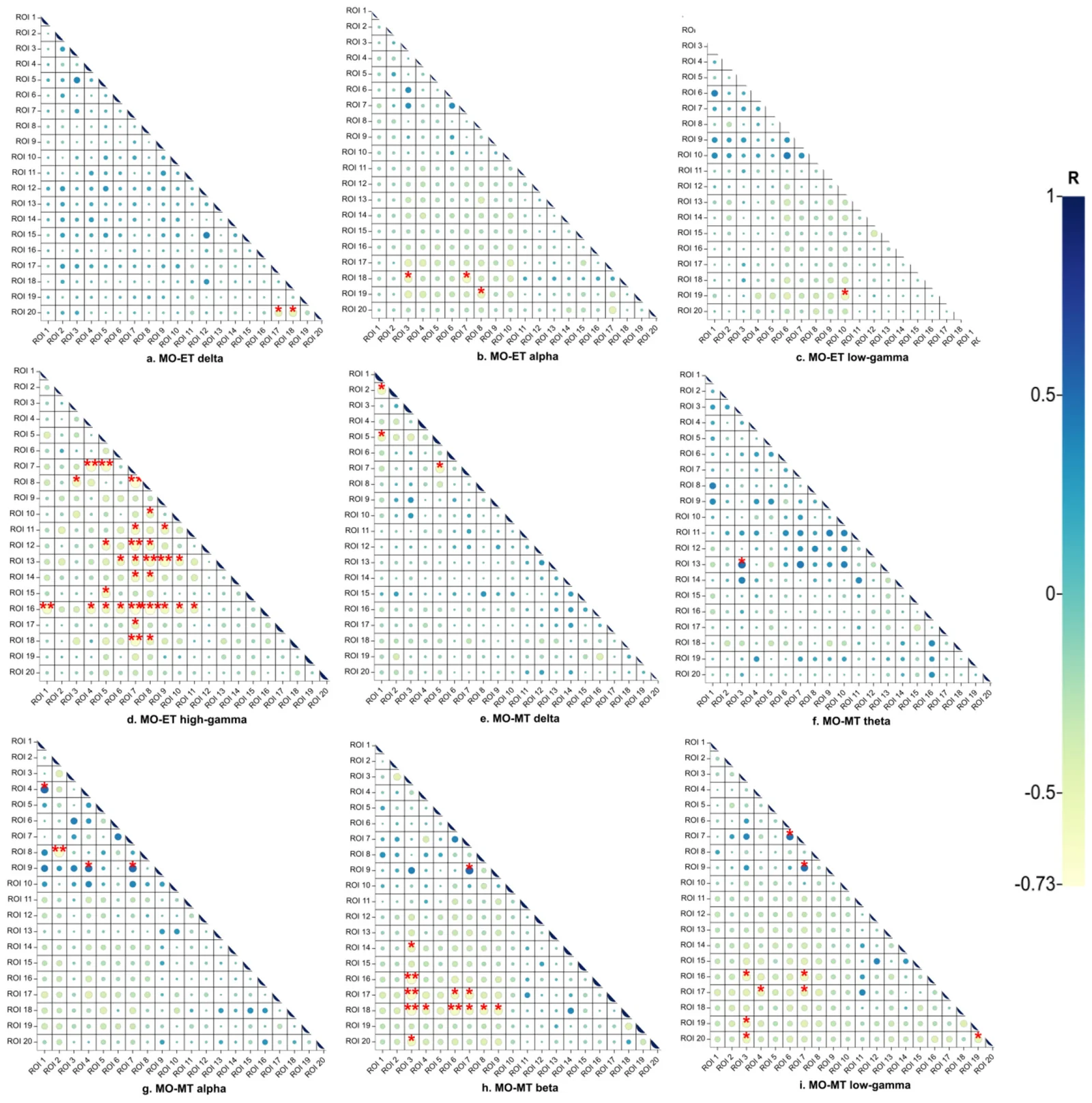
**(a)** Correlation matrix between functional connectivity in the δ band and ET motor performance in the MO group. **(b)** Correlation matrix between functional connectivity in the α band and ET motor performance in the MO group. **(c)** Correlation matrix between functional connectivity in the low-γ band and ET motor performance in the MO group. **(d)** Correlation matrix between functional connectivity in the high-γ band and ET motor performance in the MO group. **(e)** Correlation matrix between functional connectivity in the δ band and MT motor performance in the MO group. **(f)** Correlation matrix between functional connectivity in the θ band and MT motor performance in the MO group. **(g)** Correlation matrix between functional connectivity in the α band and MT motor performance in the MO group. **(h)** Correlation matrix between functional connectivity in the β band and MT motor performance in the MO group. **(i)** Correlation matrix between functional connectivity in the low-γ band and MT motor performance in the MO group. The diameter and color intensity of the circles within each cell represent the strength of the correlation; larger and darker circles indicate stronger correlations. Red asterisks *denote significant correlations. The color bar on the right represents the range of correlation coefficients (*R* values), with blue indicating positive correlations and yellow indicating negative correlations. ET, easy task; MT, medium task; MO, motor observation.

**Figure 6:**
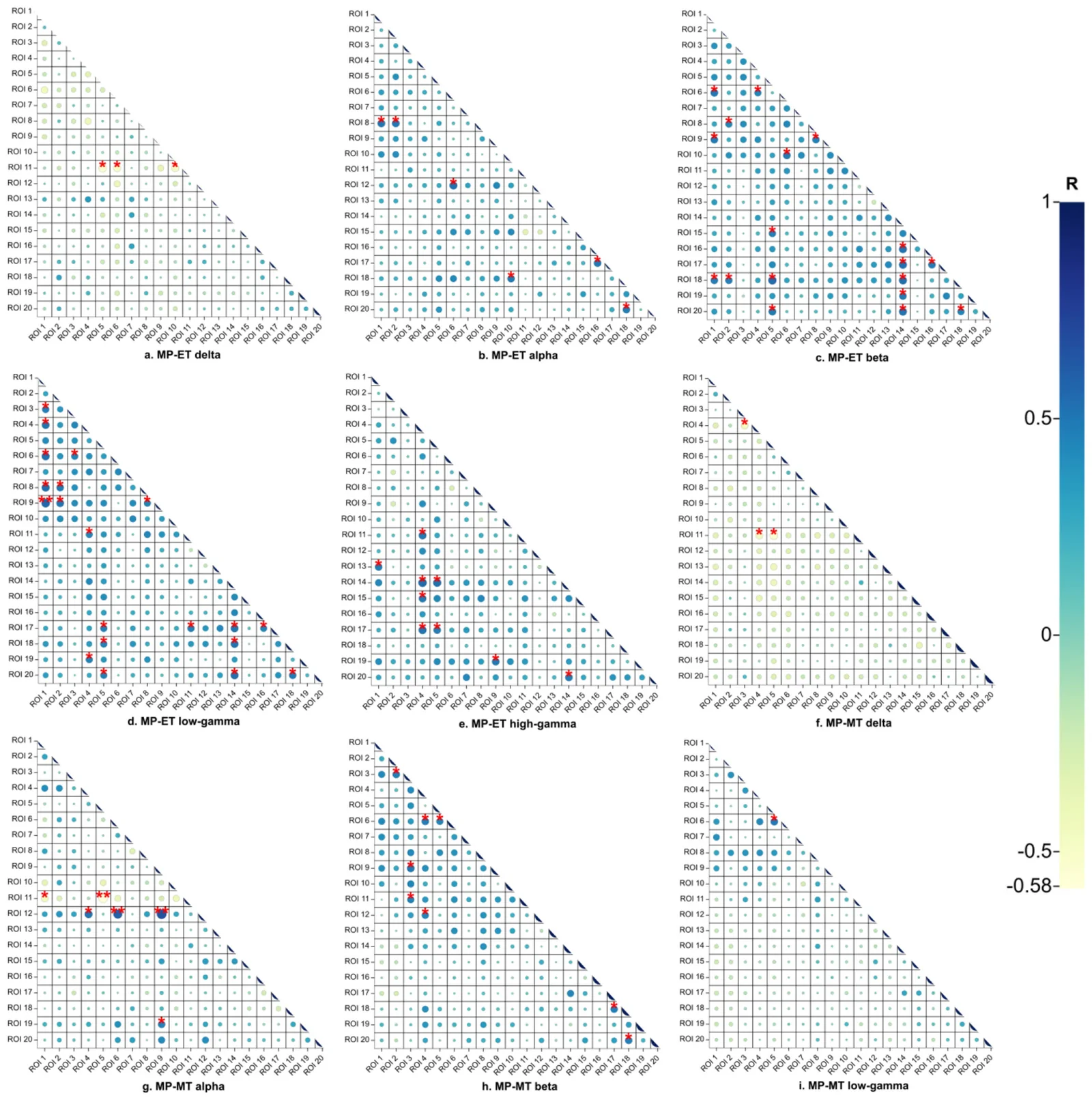
**(a)** Correlation matrix between functional connectivity in the δ band and ET motor performance in the MP group. **(b)** Correlation matrix between functional connectivity in the α band and ET motor performance in the MP group. **(c)** Correlation matrix between functional connectivity in the β band and ET motor performance in the MP group. **(d)** Correlation matrix between functional connectivity in the low-γ band and ET motor performance in the MP group. **(e)** Correlation matrix between functional connectivity in the high-γ band and ET motor performance in the MP group. **(f)** Correlation matrix between functional connectivity in the δ band and MT motor performance in the MP group. **(g)** Correlation matrix between functional connectivity in the α band and MT motor performance in the MP group. **(h)** Correlation matrix between functional connectivity in the β band and MT motor performance in the MP group. **(i)** Correlation matrix between functional connectivity in the low-γ band and MT motor performance in the MP group. The larger and darker circles indicate stronger correlations. Red asterisks *denote significant correlations. The color bar on the right represents the range of correlation coefficients (R values), with blue indicating positive correlations and yellow indicating negative correlations. ET, easy task; MT, medium task; MP, concurrent observation and physical practice.

## 5 Discussion

Overall, the most noteworthy finding was that the source functional connectivity between specific ROIs was stronger in the MP group than in the MO group. Although the two groups differed in age, the primary behavioral findings remained significant after controlling for age using ANCOVA. Therefore, the connectivity differences are interpreted primarily in relation to different learning methods, while potential age-related influences should be considered. The absence of significant connectivity changes in other ROIs within the same anatomical region does not necessarily indicate functional redundancy. Functional connectivity reflects condition-dependent interactions between specific cortical nodes rather than isolated regional functions. Therefore, selective changes in particular ROI pairs may reflect the recruitment of specific neural pathways rather than uniform changes across all regions within the same anatomical category. Moreover, source functional connectivity in MP was generally positively correlated with motor performance. These differences may be associated with the distinct learning approaches employed in this study. From an embodied learning perspective, the advantage of MP may be related to the increased bodily engagement and task integration provided by physical practice. Compared with MO, MP requires learners to actively execute observed movements while continuously integrating visual information with action-related sensory experiences, thereby creating a richer learning condition involving both observation and bodily participation. This enhanced bodily involvement may facilitate the formation of more integrated sensorimotor representations and may contribute to adaptive changes in neural connectivity during skill acquisition. These overall results align with previous studies showing that repetitive movements for several minutes can induce neural reorganization within the human motor cortex, an effect termed use-dependent plasticity (Classen et al., 1998). Furthermore, combining observation and physical practice, as in MP, has been suggested to enhance motor learning-related neural adaptations (Celnik et al., 2006).

### 5.1 Learning effects of MP compared to MO

Behavioral analysis demonstrated a significant main effect of Learning Methods, indicating that participants who learned through concurrent observation and physical practice achieved better motor performance than those who learned through observation alone. Moreover, the absence of a significant Learning Methods × Task Levels interaction suggests that this behavioral advantage was consistent across both Tai Chi tasks.

This finding is consistent with real-world physical education practice, where observation alone is often insufficient for successful motor skill acquisition. Learners often fail to retain movement sequences when attempting immediate reproduction after observation, with retention declining further over time. A similar result was reported by Xing et al. (2014) who found that feedback study is more efficient than observation study. In our study, MP likewise provided additional proprioceptive information during learning, contributing to more efficient sensorimotor integration and superior motor performance.

Although a significant main effect of Task Levels was identified, this result is not interpreted as evidence of superior learning of MT. Because the present design did not experimentally dissociate task difficulty from task order, the observed task effect cannot be unequivocally attributed to task complexity alone. Therefore, the present study focuses primarily on the robust Learning Methods effect, which directly addresses the research question concerning the effectiveness of concurrent observation and physical practice relative to observation alone.

### 5.2 Functional connectivity between lateral pre-frontal and somatosensory, lateral pre-motor areas during learning ET compared to MT

The current study showed that, in the MP group, differences between the two tasks were primarily observed in short-range functional connectivity within parietal areas; specifically, source functional connectivity between PMC & SMA and SAC during MT learning was significantly higher than during ET learning on the δ band. This finding was consistent with H2, and was consisted with previous studies demonstrating that pre-motor frontal and parietal areas guide features of more complex motor tasks (Cosottini et al., 2012), and that activation of the SAC and PMC & SMA has been associated with complex movements requiring higher attentional demands (Knaepen et al., 2015). Thus, compared with the ET, the MT may require greater attentional, sensory, and motor resources from PMC & SMA and SAC. However, given the fixed task order, the observed increase in connectivity may also reflect accumulated learning experience and task-order effects. Thus, we propose that the increased connectivity observed between PMC & SMA and SAC during MT learning in MP participants may reflect enhanced engagement of motor and sensorimotor networks under increased task demands. However, this interpretation should be considered preliminary because task difficulty was not fully dissociated from task order in the current design. This finding may indicate greater involvement of motor adaptation processes when participants encountered more demanding movement requirements. A similar result also was also found in a basketball study, which reported that lower brain activation was observed in PMC & SMA regions during slow basketball dribbling as compared to the fast-dribbling condition (Carius et al., 2020). However, these results contrast with those of Jenkins et al. (1997) who found no differential cortical activation between slow and fast finger tapping. We inferred that discrepancy may be attributable to task complexity itself, as finger tapping is considerably simpler than sport skills such as basketball and Tai Chi. This easy task represents a fundamental motor activity that humans have acquired proficiently over extended periods, leading to automated neural processing that minimizes differential brain activation between task variations.

We acknowledge that results from the aforementioned alternative speed movements studies may not be directly comparable to ours, as these studies manipulated movement execution speed, whereas our study focused on movement complexity. Nevertheless, as increased speed is directly related to the difficulty level of movement (Maruyama et al., 2021) and PMC & SMA were commonly activated areas in these studies, the results of slow vs. fast movement execution are largely analogous to the current ET and MT learning process.

### 5.3 Functional connectivity between lateral pre-frontal and somatosensory, lateral pre-motor areas during MP compared to MO

Unlike the ET–MT comparison, which showed relatively localized functional connectivity differences, the MO–MP comparison revealed more extensive differences involving both short- and long-distance source functional connectivity across several frequency bands. At the same time, we found the source functional connectivity was stronger in MP group no matter in learning ET or MT. While descriptive trends (e.g., alpha-band fluctuations in Supplementary material S3) may suggest incidental variations, our corrected statistical analyses confirm that significant group differences occur exclusively in δ, θ, and γ bands. Overall, the primary result of the between-group comparison revealed that MP exhibited greater activity in FPN (fronto-parietal network). This finding was consistent with H1. We identified a distinct network comprising FPC, M1, DLPFC and SAC. This key finding echoes previous reports, which have shown that increased connectivity within FPN is observed after a single session of short-term motor learning (Albert et al., 2009). Furthermore, heightened connectivity within the FPN is known to correlate with improved motor performance. During the early consolidation phase, it appears that greater connectivity among brain areas involved in motor acquisition translates into superior learning performance (Sami et al., 2014). Honda et al. (1998) reported that decreased reaction times were associated with increased activity in M1 and SMA. Sun et al. (2007) highlighted an increased interaction between the frontal network and the motor network during early learning, particularly as subjects continued to enhance their accuracy. Therefore, we posit that the enhanced FPN connectivity may reflect greater involvement of motor learning-related processes and, consequently, better learning performance.

#### 5.3.1 Comparison between MP and MO in ET

During ET learning, MP showed a higher connectivity between FPC and M1, DLPFC and SAC in the high-γ frequency band, suggesting that motor learning-related information is distributed in networks associated with both motor and higher order cognitive regions. This result is in line with Sun's study, which found that the connectivity between the motor network and higher order cognitive regions is enhanced during early learning (Sami et al., 2014). M1 is known to be essential for planning, preparation and execution of movements (Leff et al., 2011). Sami et al. demonstrated the role of the FPN (fronto-parietal network, FPN) in explicit learning, and identified bilateral activation in both the parietal and pre-motor regions. This network is thought to represent an engram of the extra procedural learning skill acquired through explicit acquisition (Maruyama et al., 2021). In addition, a similar M1 and FPC network was also discovered by other researchers. Maruyama et al. suggested that the M1-centered network with the FPN represents the formation of declarative procedural skills (Carius et al., 2020). Therefore, we conclude that higher FPN connectivity in MP might reflect advanced procedural learning skill.

The PPC (posterior parietal cortex, PPC), which encompasses the SAC, is necessary for early and late learning phases. The PFC (prefrontal cortex, PFC), which includes the DLPFC, is primarily involved in early learning phases (Dahms et al., 2020). The PFC processes sensory input, motor output, and working memory, whereas the PPC processes spatial-sequential components (Carius et al., 2020). The DLPFC-SAC connection exists in the similar anatomical region. Loayza et al. (2011) found that somesthetic egocentric spatial perception is related to the connectivity between the right superior parietal lobe, DLPFC and lingual gyrus. This finding suggests concurrent activation of SAC and DLPFC, supporting multi-information flow in closed motor skill learning—a process involving multivariate perceptual coordination.

In our study, the M1 was only found to be connected with the FPC. Why was M1 less connected with other areas in this study, given its importance for motor behaviors? We believe it could be explained with the following reasons. Firstly, M1 and PPC are critical hubs for the late motor sequence learning phase since these areas contribute to the delayed recall of learned motor sequences (Penhune and Doyon, 2002). Evidence suggests that the M1 integrates the accumulated information processed by the PPC in motor sequence learning in the later phases of learning (Carius et al., 2020). That is to say, the later phase of motor learning involves retrieving the learned sequences acquired during the early learning phase. Since the present study focused on the early stage of learning, which may involve limited retrieval of consolidated motor sequences, weaker involvement of M1-related connectivity with other regions may reflect the characteristics of early learning rather than reduced functional importance of M1. Secondly, M1 is known as to encode integrated spatiotemporal information (Kornysheva and Diedrichsen, 2014), which is primarily processed in temporal and occipital areas. It is possible that M1 might have been connected with these areas, but our data did not cover these areas. We acknowledge this as a limitation of the study: ROI selection was restricted to frontal and parietal areas, and did not encompass the whole brain. Third, the M1 contributes primarily to the execution of gait modifications (Drew et al., 2008), while the PMC & SMA, and PFC are more activated in the motor planning and coordination (Sahyoun et al., 2004). This might explain why the M1 is less connected than the PMC & SMA during Tai Chi learning, which requires planning and cooperation of multi-sensory inputs and body part (e.g., eye-upper limb-lower limb coordination).

#### 5.3.2 Comparison between MP and MO in MT

In the process of learning MT, MP showed a higher connectivity between DLPFC and FPC, between PMC & SMA and SAC, between FPC and SAC, and within SAC in the δ frequency band, within FPC in the θ frequency band, between FPC and SAC, and within SAC in the low-γ frequency band. The DLPFC, a task positive region, is one of the key regions of the executive control network, playing significant roles in top-down cognitive control processes (CCN) (Liu et al., 2018). Studies have indicated that repetitive transcranial magnetic stimulation (rTMS) and transcranial direct current stimulation (tDCS) on DLPFC are capable of enhancing memory-guided responses in the visuospatial working memory tasks (Brunoni and Vanderhasselt, 2014). These findings further verified one of the important roles of the DLPFC in the Tai Chi learning process: memorizing Tai Chi-specific movements and sequences. A similar connection in DLPFC-FPC related areas was found in another Tai Chi research. Tao et al. (2017) proved that Tai Chi practice significantly modulates the rsFC (rest state functional connectivity, rsFC) between the CCN and the superior frontal gyrus. While greater connectivity of the DLPFC predicts better memory performance (Grady et al., 2010), we suggest that the enhanced connectivity in the pre-frontal area indicates a better motor memory capacity in MP.

Regarding alteration in PMC & SMA-SAC connection, our results were in line with the previous studies showing that the early motor skill learning stage is characterized by increased functional connectivity in the motor cortex–sensorimotor circuit (Chen et al., 2022). These areas together are known as a pathway of sensory and motor interactions. The posterior parietal cortex (PPC), which encompasses SAC, is a multisensory integration area with abundant projections to motor areas (Koch et al., 2007) and is involved in motor planning (Koch et al., 2008). The SAC codes relative body position and spatial information (Gentile et al., 2011), and transforms multisensory inputs into a common coordinate system through visual, auditory, and proprioceptive integration (Lewis et al., 2011). This area is known to send intrahemispheric inputs to the arm and hand representations in motor-related areas in humans (Premji et al., 2011). A sensorimotor network (SMN) related study has shown that both somatosensory (postcentral) and motor (putamen and precentral gyrus) areas are involved in pre-mediated state of readiness to perform or coordinate a motor task (Chenji et al., 2016). The activation of the PMC & SMA could be explained by their role in execution and observation of object-related hand movements. This corresponds to the fact that Tai Chi involves many hand movements, such as “pushing palm” and “hook grasp,” which were frequently mentioned in ET and MT. Studies on monkeys indicated the parieto-frontal circuit, which is comprised of the connections between parietal areas and ventral pre-motor cortex (Murata et al., 1997), provides the anatomical basis for the transformation of sensory information into actions (Rizzolatti and Luppino, 2001). SMA has also been shown to participate in encoding aspects related to preparation and execution during voluntary and triggered sequential movements of the upper limbs (Dai et al., 2001), coordination between arm and leg (Debaere et al., 2001). Pre-motor and posterior parietal areas are suggested to mediate perception aspects regarding spatial distance estimation and coding of spatial locations relative to body parts, particularly in egocentric frame-referencing (Committeri et al., 2004). This function aligns with the Tai Chi teaching instructions such as “Keep your hands together toward the midline of your body,” “Point your fingertips to the tip of your nose,” “Align right hand to the inside of the left elbow.” Given SMA involvement in complex sequence movements, the regional strengthening may be indicative of late motor consolidation (Maruyama et al., 2021). We suggest the increased PPC connectivity in this study demonstrates deeper learning information processing, i.e., better motor consolidation in MP.

During early learning of MT, the connectivity between the motor network and higher-order cognitive regions (FPC-SAC) is enhanced. It has been verified that FPC and SAC contributes to executive function and fine motor skills and are strongly associated with cognitive and motor function (Koini et al., 2018). Maruyama et al. suggest that learning-related information is distributed in networks associated with both motor and executive controls, evidenced by an increment of the preparatory activity in regions that include the SMN and FPN (Carius et al., 2020). The FPN controls coordinated behavior in a rapid, accurate, and flexible goal-driven manner (Marek and Dosenbach, 2018). Thus, the higher connectivity between FPC and SAC in MP is probably related to extra procedural learning skill in a relatively later learning stage (Maruyama et al., 2021). These cross-region neural pathways integrate functions that are necessary for motor learning and play a vital and stable role in sport skills acquisition.

### 5.4 Learning-method-dependent relationships between source functional connectivity and motor performance

The correlation results were consistent with H3. In the MP group, stronger functional connectivity was positively associated with better motor performance, whereas several negative correlations were observed in the MO group. These findings suggest that the neural networks supporting motor learning may differ between concurrent observation and physical practice and observation-only learning. The positive association observed in the MP group is consistent with previous reports showing that stronger functional connectivity is associated with superior motor learning performance (Heitger et al., 2012). However, the divergent correlation patterns in MO and MP remain unclear. Because participants in the MO group relied solely on visual observation without physical execution, the absence of proprioceptive feedback may have resulted in different patterns of neural resource allocation compared with MP. The MO condition may rely more heavily on visual and cognitive processing because it lacks direct bodily involvement. However, this interpretation remains speculative, and differences in cognitive demands, attentional strategies, or task engagement may also contribute to the observed correlation patterns. In contrast, participants in the MP group received additional proprioceptive feedback through physical execution, which may have contributed to different patterns of neural processing during motor learning. Nevertheless, alternative explanations should also be considered, including compensatory recruitment, differences in task engagement, or individual variations in the strategies used during observational learning.

### 5.5 Limitations of the study and future studies

The first limitation of this study concerns the fixed order of task presentation. This design was intentionally adopted to examine neural adaptations during progressive difficulty-ascending motor learning, reduce timing variability associated with manual experimental procedures, and facilitate participants' gradual adaptation to the experimental protocol, particularly for individuals with no prior Tai Chi experience. Nevertheless, this design inevitably introduces a potential confounding effect between task difficulty and task order. Consequently, the observed differences between ET and MT cannot be attributed solely to differences in task complexity. They may also reflect familiarization with the experimental environment, cumulative learning experience across the session, or fatigue associated with completing the later task. Therefore, the present findings should be interpreted cautiously. Rather than providing definitive evidence for difficulty-dependent neuroplasticity, the observed neural differences may represent the combined influence of increasing task demands and task-order-related learning effects. This limitation is particularly relevant to the interpretation of the behavioral results. The higher motor performance observed during MT should therefore not be interpreted as evidence that participants learned the more difficult task more successfully. Instead, this finding may reflect the combined influence of task order, increasing familiarity with the experimental procedure, and cumulative practice effects. Future studies should employ counterbalanced task orders or randomized task presentation to disentangle the independent contributions of task complexity and task order.

Another limitation is that the present design does not isolate the effects of physical practice from action observation. Participants in the MP group practiced while observing the instructional video; therefore, the observed advantage is the effect of embodied action observation training.

This study was also limited by the age difference between groups. Although age-controlled ANCOVAs demonstrated that the behavioral advantage of MP remained significant after accounting for age, the current EEG connectivity analyses were not performed with age as a covariate. Therefore, potential age-related influences on neural connectivity cannot be completely excluded. Future studies with age-matched participants or larger samples allowing covariate-based neuroimaging analyses are warranted.

Additionally, another limitation concerns the potential influence of movement-related artifacts on EEG connectivity measures. Since participants in the MP condition performed Tai Chi movements during EEG acquisition, whereas participants in the MO condition remained relatively still, the possibility that residual motion-related artifacts contributed to between-group differences cannot be completely excluded. Although we minimized excessive body movements and applied ICA-based artifact correction and artifact rejection procedures, these approaches may not fully eliminate all movement-related noise in mobile EEG recordings. Therefore, the enhanced functional connectivity observed in the MP condition should be interpreted cautiously, as it may reflect both genuine learning-related neural processes and residual movement-related contributions. Future studies could combine EEG with synchronized motion capture systems, inertial sensors, or advanced artifact correction algorithms specifically designed for movement-rich paradigms to further validate these findings.

Future researchers should consider these limitations and address them in subsequent studies. For example, computerized timing programs should be designed based on experiment tasks, non-beginners should be recruited to compare pure practice with observation, and longitudinal experiments including retention tests of Tai Chi learning should be conducted. Additionally, future research should address themes relevant to authentic physical education settings, such as sports teaching methods (e.g., demonstration, explanation, and error correction), online vs. face-to-face learning, and optimal timing for employing different learning methods, thereby promoting evidence-based sports pedagogy and efficient learning.

## 6 Conclusion and implications

In physical education settings, combining observation with physical practice is commonly considered beneficial for acquiring motor skills. Does concurrent observation and physical practice truly outweigh mere observation during sport skill learning, and if so, how? Based on this question, this paper investigated the source functional connectivity of MP and MO, ET and MT, evaluated their motor performance, and analyzed the correlation between learning methods and motor performance. Behavioral and EEG data indicated that MP prevailed over MO; specifically, MP manifested higher motor performance and stronger source functional connectivity in several frequency bands, both between and within frontal and parietal areas. Furthermore, correlation analysis demonstrated that the source functional connectivity in MP was significantly positively associated with Tai Chi motor performance. In terms of differences between task difficulty, MT was associated with stronger source functional connectivity than ET only in the MP group.

The current study proposed a learning method framework for physical education, namely, concurrent observation and physical practice. This framework differs substantially from those in other classroom disciplines (e.g., history), which primarily employ observation-centered learning methods. This study outlined the essential place of MP in novel closed motor skill learning methods. Consequently, this study suggests that physical education teachers should emphasize the importance of MP, ensure its integration as an indispensable component of instruction, and guide students to employ MP during sport skill learning. In addition to MP, observation is recommended in conjunction with MP (e.g., observation following practice), as a study demonstrated that observing movements identical to those just practiced benefits subsequent motor performance (Fadiga et al., 1995). Furthermore, when practicing higher-difficulty motor skills, physical education teachers and students should exercise patience, as such tasks demand greater cognitive and neural resources.

## Data Availability

The raw data supporting the conclusions of this article will be made available by the authors, without undue reservation.
