# Supplementary material for: Concurrent observation and physical practice facilitates early closed motor skill learning: evidence from EEG source functional connectivity and behavioral performance
Source: Front Psychol. 2026 Sep 18;17:1865823. doi: 10.3389/fpsyg.2026.1865823 (PMC13630685; doi:10.3389/fpsyg.2026.1865823)
Supplement: Supplementary file 1 [file Table_1.DOCX]

Supplementary Material S4

**Ethical Compliance Protocols**

**Scientific Justification:**

The study’s primary objective was to investigate neuroplastic mechanisms underlying motor learning (MO vs. MP) in an ecologically valid Tai Chi learning paradigm. This required:

(1) Novice participants to eliminate prior experience confounds;

(2) EEG recordings during active movement to capture real-time neural dynamics;

(3) Group comparisons to address our core hypothesis about neural efficiency.

**Ethical risk mitigation measures included:**

Psychological Protection: No performance pressure was imposed; participants were informed that task performance would not affect compensation.

Benefit-Risk Ratio Justification: Beyond minimal physical risk, the ethical justification for participant involvement rests on the study’s potential scientific contribution. By clarifying the distinct neurocognitive pathways underlying observation versus practice in closed-skill acquisition, the findings will inform evidence-based pedagogies for Tai Chi and other closed motor skill courses in Chinese universities. This benefit outweighs the inconvenience of a single 30-minute experimental session and the inherent safety of EEG as a non-invasive neuroimaging technique.

**All participants received detailed risk-benefit documentation prior to consent, including:**

Potential discomforts: EEG cap adjustment (mild pressure);

Benefits: No direct therapeutic benefit, but contribution to sports pedagogy optimization, compensation and souvenirs;

Alternative options: Voluntary withdrawal at any stage without penalty.

**The EEG Data Processing Pipeline**

Data Format Conversion:

Raw data (TMSi SAGA format) → converted to EEGLAB-compatible .set files.

Channel Localization:

Electrodes assigned to standard 10-20 positions.

Redundant Electrodes Removal:

Non-cephalic channels (e.g., reference electrodes) removed, retaining 62 scalp channels.

Filtering:

low pass filter: 1 Hz; high pass filter: 100 Hz; Notch: 48–52 Hz.

Downsampling:

Original 1000 Hz → 500 Hz.

Epoch Segmentation:

Continuous data divided into non-overlapping 2-second intervals.

Bad Epoch/Channel Handling:

Epoch Rejection: Exclusion of bad epochs.

Channel Interpolation: Spherical spline interpolation for bad electrodes.

ICA Decomposition:

Extended Infomax ICA (EEGLAB runica) on cleaned continuous data.

Artifact Component Removal:

Outlier Rejection:

Final epoch screening: Exclude epochs exceeding 100 µV.

Re-referencing:

Average reference applied post-cleaning to avoid reference bias.

Manual Quality Check:

Visual inspection of cleaned data by two trained raters.

**The Workflow of Source-Based Functional Connectivity Analysis with sLORETA**

**Source Localization and ROI Definition:**

1. Cortical Source Reconstruction:

Scalp EEG signals from 62 electrodes were input to the sLORETA algorithm to estimate cortical source activity.

sLORETA solves the electromagnetic inverse problem by computing the standardized current density distribution across 6239 gray matter voxels (5mm×5mm×5mm voxels).

2. ROI Selection:

20 cortical regions of interest (ROIs) were selected based on their established roles in motor learning.

For each ROI, voxel-wise current density time-series within the anatomical mask were averaged to generate a single ROI-level source signal.

**Functional Connectivity Analysis:**

1. Nonlinear Lagged Connectivity (ROInonLinLagConn):

Connectivity was computed between source-level ROI pairs (not scalp electrodes) using the ROInonLinLagConn metric. Surface electrodes were used only for inverse modeling, not for direct connectivity computation.

2. Statistical Analysis:

Group-level comparisons (MO vs MP) were performed for each ROI-pair and frequency band (δ, θ, α, β, low-γ, high-γ).

Significance was assessed via 5000 permutation tests (SnPM correction).

**The Correlation Calculation Process**

1.Data Structuring:

Source functional connectivity matrices were computed for each participant and frequency band using sLORETA, resulting in 20×20 connectivity matrices.

For each learning method (MO/MP) and task (ET/MT), connectivity matrices across participants were concatenated into a 3D array of dimensions.

Motor performance scores were stored as a vector.

2. Matrix-to-Vector Conversion:

The 3D connectivity array was reshaped into a 2D matrix, where each row represents a unique ROI-pair, and each column represents a participant.

3. Pearson Correlation:

For each ROI-pair, the correlation between connectivity strength (across subjects) and motor performance scores was computed using MATLAB’s “corr” function:

[r, p] = corr(conn_vector, behavior_scores, ‘Rows', 'complete');

This generated correlation coefficients (r) and uncorrected p values per frequency band.

4. Multiple Comparisons Correction:

False Discovery Rate (FDR) correction was applied to all ROI-pair comparisons within each frequency band (“fdr_bh” function).
