# Supplementary material for: Concurrent observation and physical practice facilitates early closed motor skill learning: evidence from EEG source functional connectivity and behavioral performance
Source: Front Psychol. 2026 Sep 18;17:1865823. doi: 10.3389/fpsyg.2026.1865823 (PMC13630685; doi:10.3389/fpsyg.2026.1865823)
Supplement: Supplementary file 2 [file Table_2.DOCX]

Supplementary Material S1

Fifteen martial arts experts rated the 24 Tai Chi motions. These experts were qualified to evaluate and rank the 24-Style Tai Chi, providing a comprehensive basis for material selection. Demographic information about the martial arts experts is detailed in Table 1. The experts first assessed the difficulty of each Tai Chi motion and subsequently reviewed the classification table created by the authors. The classification was conducted under the guidance of Gentile’s motion taxonomy [^[1]^]. According to Gentile’s taxonomy and the motor characteristics of 24-Style Tai Chi, 24-Style Tai Chi could be categorized into 3 types: (1) body steadiness (relatively stationary body), no manipulation, fixed regulatory conditions, no intertrial variability; (2) body movement (primarily horizontal plane), no manipulation, fixed regulatory conditions, no intertrial variability; (3) body movement (primarily vertical plane), no manipulation, fixed regulatory conditions, no intertrial variability. The selecting criteria for the movements used in our experiment materials were: a. Motions rated as easy and medium difficulty; b. Motions belonging to the first and second classifications, as the third type were found to induce more body imbalance in preliminary experiments, leading to increased motor artifacts. Movements that are eligible were chosen for optimal data quality.

Table 1. Demographic information of the martial arts experts

| Characteristics/Level | | n | % or Mean (SD) |
| --- | --- | --- | --- |
| Age | - | 15 | 35.47±13.95 |
| Working years (research/ teaching/ training) | - | 15 | 13.20±8.10 |
| Gender | Male | 8 | 53.33 |
|  | Female | 7 | 46.67 |
| Work/ study organization | Universities | 14 | 93.30 |
|  | Secondary school | 1 | 6.70 |
| Occupation | Teacher | 9 | 60.00 |
|  | Student athletes | 6 | 40.00 |
| Professional title | Professor | 3 | 20.00 |
|  | Associate professor | 3 | 20.00 |
|  | Lecturer | 3 | 20.00 |
| Martial arts level | Master | 3 | 20.00 |
|  | First grade | 4 | 26.70 |
|  | Second grade | 1 | 6.70 |

**24-Style Tai Chi Performance Scoring Criteria**

The following standardized scoring rubric was used independently by all three judges during behavioral evaluation.

**1. Excellent (90–100 points):**

- Movements strictly adhere to standardized form; technique is precise.
- Smooth energy flow (Jin Li) with clear void-solid (Xu-Shi) differentiation.
- Seamless hand-eye coordination.
- Executes the entire routine proficiently without errors.

**2. Good (80–89 points):**

- Movements largely conform to standardized form; technique is clear.
- Energy flow (Jin Li) is relatively smooth.
- Demonstrates hand-eye coordination.
- Completes the routine competently with minor imperfections.

**3. Average (70–79 points):**

- Movements are generally correct.
- Technique is relatively clear.
- Completes the routine with occasional technical inconsistencies.

**4. Pass (60–69 points):**

- Movements contain no major errors; technique is relatively clear.
- Despite brief memory lapses and minor incoordination during performance, movement rigidity is not significantly apparent.
- Can independently complete the entire routine (with ≤2 memory lapses).

**5. Fail (<60 points):**

- Movements are non-standard; technique is unclear.
- Lacks familiarity with the routine.
- Unable to perform independently.

Since the 24-form Tai Chi Chuan is rooted in traditional Chinese martial arts culture, certain specialized terms may not be fully captured in translation. Therefore, the original Chinese text is provided here for reference.

**24式太极拳评分标准**

| 评价标准 | 等级 | 分数 |
| --- | --- | --- |
| 动作规范，方法清楚，劲力顺达，虚实分明，手眼协调配合，熟练完成套路。 | 优秀 | 90分以上 |
| 动作规范，方法清楚，劲力比较顺达，手眼有配合，能较熟练的完成套路。 | 良好 | 80 - 89分 |
| 动作比较规范，方法比较清楚，能较为熟练地完成套路。 | 中等 | 70 - 79分 |
| 动作无大错误，方法比较清楚，演练时虽有短暂遗忘和动作不协调现象，但动作僵化不是很明显，能独立完成套路（遗忘不超过2次）。 | 及格 | 60 - 69分 |
| 动作不规范，方法不清楚，套路不熟练，无法独立完成套路。 | 不及格 | 60分以下 |

1. [] Gentile, A. M. (2000). Skill Acquisition: Action, movement, and neuromotor processes. In J. H. Carr, & R. B. Shepherd (Eds.), Movement science: Foundations for physical therapy (2nd ed., pp. 111–187). Rockville, MD: Aspen.
